# Object Tracking in RGB-T Videos Using Modal-Aware Attention Network and Competitive Learning

**DOI:** 10.3390/s20020393

**Published:** 2020-01-10

**Authors:** Hui Zhang, Lei Zhang, Li Zhuo, Jing Zhang

**Affiliations:** 1 Faculty of Information Technology, Beijing University of Technology, Beijing 100124, China; zhanglei@emails.bjut.edu.cn (L.Z.); zhuoli@bjut.edu.cn (L.Z.); zhj@bjut.edu.cn (J.Z.); 2 Beijing Key Laboratory of Computational Intelligence and Intelligent System, Beijing University of Technology, Beijing 100124, China

**Keywords:** RGB-T object tracking, cross-modal data fusion, modal-aware attention network, competitive learning

## Abstract

Object tracking in RGB-thermal (RGB-T) videos is increasingly used in many fields due to the all-weather and all-day working capability of the dual-modality imaging system, as well as the rapid development of low-cost and miniaturized infrared camera technology. However, it is still very challenging to effectively fuse dual-modality information to build a robust RGB-T tracker. In this paper, an RGB-T object tracking algorithm based on a modal-aware attention network and competitive learning (MaCNet) is proposed, which includes a feature extraction network, modal-aware attention network, and classification network. The feature extraction network adopts the form of a two-stream network to extract features from each modality image. The modal-aware attention network integrates the original data, establishes an attention model that characterizes the importance of different feature layers, and then guides the feature fusion to enhance the information interaction between modalities. The classification network constructs a modality-egoistic loss function through three parallel binary classifiers acting on the RGB branch, the thermal infrared branch, and the fusion branch, respectively. Guided by the training strategy of competitive learning, the entire network is fine-tuned in the direction of the optimal fusion of the dual modalities. Extensive experiments on several publicly available RGB-T datasets show that our tracker has superior performance compared to other latest RGB-T and RGB tracking approaches.

## 1. Introduction

Video object tracking is a basic task in data processing of imaging sensors. It is widely used in video surveillance, autonomous vehicles, robots, and other fields. In recent years, with the advancement of imaging sensor technology, imaging devices are developing rapidly towards the direction of low cost and miniaturization, and these cameras working in different spectral bands are increasingly popular. Among them, the uncooled infrared camera has a powerful complement and expansion of the widely deployed visible spectrum imaging system due to its ability to work in all-weather and all-day scenarios. Correspondingly, the demand for automatic analysis and processing based on visible-infrared dual-modality data is proliferated, and object tracking based on RGB-thermal (RGB-T) data has also received widespread attention as one of its core technologies.

The object tracking problem of RGB-T is an extension of the traditional visual tracking task, that is, given the initial position state of the target, the RGB and thermal infrared image are comprehensively used to continuously estimate the target position in subsequent scenes. In recent years, several works have been carried out on this RGB-T tracking research, and representative approaches are roughly divided into two categories: Tracking based on traditional manual features [1,2,3,4,5,6,7] and tracking based on deep learning [8,9,10,11].The former category is mostly based on theoretical frameworks such as sparse representation [2,3,4,5], correlation filtering [6], Bayesian filtering [7], and uses hand-crafted textures or local features to construct cross-modal object appearance model and state estimation methods. The latter class builds the effective model of modeling targets from massive data by exploring the powerful feature representation capabilities of deep neural networks. This kind of work has shown great development potential in many related fields due to the end-to-end and data-driven advantages of deep networks.

Generally speaking, the existing works have made great progress in the fusion and utilization of dual-modality data. However, in the face of complicated application scenarios, there are still the following technical challenges: (1) although infrared imaging is not affected by environmental factors such as illumination, rain, smoke, and haze, it is limited by the inherent resolution of the infrared band, atmospheric absorption, and scattering during propagation, as well as the imaging characteristics of the sensor. The signal-to-noise ratio, contrast, and resolution of the infrared image are worse than those of the visible spectrum image. How to make full use of the heterogeneity between two modalities, effectively extract features and construct modality complementary representation is the basis for obtaining a robust tracker. (2) Based on the above feature representation, designing a deep network framework for video object tracking and its learning strategy, and establish a scene-adaptive fusion method for cross-modal features to overcome the limitations of single modality and improve the tracker performance.

Motivated by the aforementioned issues, this paper proposes an RGB-T object tracking method based on a modal-aware attention network and competitive learning (MaCNet) within the deep learning and tracking-by-detection framework. The proposed tracking network includes three parts: a feature extraction network, modal-aware attention network, and a classification network. The feature extraction network adopts the form of a two-stream network, which extracts hierarchical features for the two modality images separately. The modal-aware attention network explores the attention parameters that characterize the importance of features at each layer, and effectively guides the feature fusion, thereby enhancing cross-modal information interaction. After the feature extraction network, the classification network utilizes three parallel classification layers acting on the RGB branch, the thermal infrared branch, and the fusion branch respectively, and builds a modality-egoistic loss function with competitive learning to guide the entire network optimization towards the dual-modality cooperation and complementarity. We evaluated the proposed tracker on two RGB-T tracking benchmark datasets: GTOT [12] and RGBT234 [13]. Our method shows favorable performance against the state-of-the-art approaches.

The main contributions of this paper are summarized as follows: firstly, a novel modal-aware attention network is proposed, which can perceive the importance of each modality and guide the adaptive fusion of dual-modality features on multiple feature layers. Secondly, based on competitive learning, a model training approach is introduced to improve the performance of modality fusion. Thirdly, a deep tracker MaCNet is constructed for RGB-T video tracking based on the above two points, which effectively exploits the complementary characteristics of heterogeneous modality data. In addition, the proposed method is a universal framework and can be easily extended to other multi-modality scenarios.

## 2. Related Work

In this section, according to the relevance of our work, we briefly review three aspects: RGB-T tracking, attention mechanisms in visual tasks, and competitive learning.

### 2.1. RGB-T Tracking

Video object tracking is a basic task in computer vision and video processing field. A comprehensive review of tracking methods for a single imaging spectrum can be found in [14,15,16]. The state-of-the-art methods can be divided into two broad categories: correlation filtering based methods and deep learning-based methods. The former is based on the two-dimensional correlation filtering operation, which makes the tracker achieve a good compromise between real-time and robustness with high computing efficiency and algorithm robustness [17,18,19,20,21,22]. The latter constructs deep tracker, which uses the powerful feature representation capabilities of deep neural networks and data-driven model construction methods, has a significant improvement in robustness compared to the previous methods. In addition, with the continuous optimization of deep network structures and model-solving algorithms, such methods are increasingly showing performance advantages, and typical works are [23,24,25,26].

Along with the improvement of the above-mentioned methods, the corresponding performance of RGB-T trackers has been continuously upgraded. Representative works [2,6,7,8,9,10,11] are based on sparse representation, correlation filtering, and deep learning. Li et al. [2] proposed a cross-modal manifold sorting algorithm, which solved the influence of background clutter during the tracking process. Wang et al. [6] proposed a soft-consistent correlation filters dedicated to RGB-T data, and achieved real-time tracking by using fast Fourier transform. Wu et al. [7] integrated candidate objects from different sources into a one-dimensional vector and forms a sparse representation in the object template space, then combines the sparse solution with a particle filtering framework to obtain an improvement in RGB-T tracking performance. In addition, Li et al. [8] proposed a convolutional neural network architecture, which integrated the two-stream network and fusion network to achieve the adaptive fusion of different modality data. Zhu et al. [9] proposed a recursive strategy to densely aggregate deep features and prune the aggregated features of each modality in a cooperative manner, which can effectively reduce redundancy and noise incurred by the feature representation. Lu et al. [10] proposed a multi-adapter convolutional network, taking full advantage of the potential value of shared information between modalities and instance-aware information. Different from general visual tracking methods, these methods focus more on exploring information sharing and cooperation of different modalities. We build a cross-modal object description model to obtain an extension of single-modality tracking performance and broaden the application scenarios of video tracking.

### 2.2. Attention Mechanisms in Vision Tasks

The attention mechanism in computer vision [27,28,29,30,31] is mainly for the algorithm of learning how to focus on the regions of interest and it plays an increasingly important role in solving many vision tasks. In recent years, most of the work of combining visual attention and deep learning is to form attention parameters by introducing a feature mask. The principle of this mask is to identify informative features in the image data through another layer with new weights. These obtained attention parameters will be applied to different feature mapping layers after training so that the deep neural network can autonomously focus attention points and highlight the target subject of interest in different feature layers. Wang et al. [28] improved the classification performance of the network by introducing the attention module at different levels, demonstrating that attention not only enables the operation to focus on a particular region, but also enhances the features of that region. Zhang et al. [29] added a channel attention to the residual network, indicating that the network characteristics captured by different channels are different, thus using these differences to improve the accuracy of image super-resolution. Zhu et al. [30] proposed a new spatio–temporal attention strategy in the visual tracking task, which fully models the impact of the previous *N* frames on the current frame by weighting the previous *N* frames. Our method is inspired by the above ideas, but unlike previous work, we explore the attention of each modality in order to estimate the importance of the corresponding features based on real scene data.

### 2.3. Competitive Learning

Competitive learning is the common learning strategy in Ad hoc network [32,33,34,35]. It encourages all units in the network group to compete for the right to respond to external stimulus patterns. The connection right of the winner unit changes in a direction that is more favorable to the competition of this stimulus patterns. At present, the most popular generative adversarial nets (GANs) [36] is a kind of deep network architecture based on competitive learning. It is used to obtain a data generation model whose data distribution is consistent with or as close as possible to the statistical distribution of the observed space. The more general form of competitive learning is not only allowing a single winner to appear, but also allowing multiple winners to appear, and the learning takes place on the connection weight of each unit in the winner set.

Competitive learning for deep neural networks usually adopts a two-stream architecture for implementation. The representative GANs networks [36,37,38] employ a two-branch form that includes generator and discriminator. The typical works are as follows. Zhao et al. [37] proposed an energy-based generative adversarial network, using the discriminator in GANs as an energy function, and training a single-scale network architecture to generate high-resolution images. Zhong et al. [38] employ CycleGAN to complete the style transfer between different camera lenses, thereby solving the problem of data scarcity in personal identification. Unlike previous work, our goal is to apply competitive learning to the field of multi-modality object tracking, so that the result of multi-modality fusion is better than the result of each single-modality branch. To our knowledge, this is the first time that competitive learning is applied to the RGB-T tracking task.

## 3. Proposed Approach

In this section, we first describe the proposed network architecture for RGB-T tracker in detail, and then introduce the two main parts of the network, including the modal-aware attention network and the modal competitive learning algorithm.

### 3.1. Tracker Architecture

The RGB and thermal infrared images captured by two cameras with aligned field-of-view (FOV) reflect the amount of radiation received by the sensors in different bands of the same scene. Correspondingly, the images of two modalities share some low-level and semantic information, and at the same time, they present a lot of heterogeneous scene details based on their spectral perspective. Furthermore, in the real-world application of video tracking, the two modalities often play different roles in different challenging scenarios. For example, when there is insufficient illumination at night, the thermal infrared modality tends to capture the position and motion of the target more easily, while in low resolution, the visible spectrum modality often provides more valuable information. Therefore, it is essential for RGB-T tracking to effectively mine and use each modality feature in different scenarios to build a cooperative and complementary cross-modal object representation.

**Figure 1:**
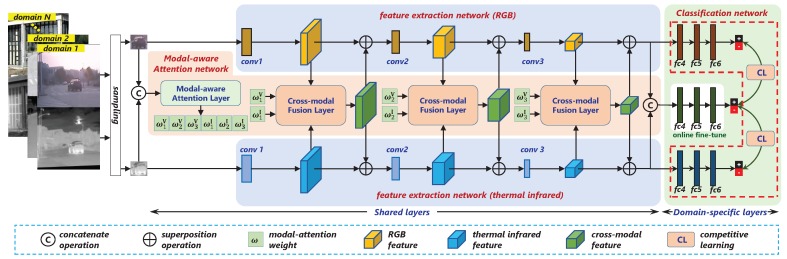
The overall architectural diagram of the proposed algorithm. Part of the network marked with dotted lines is only used for offline competition training.

To this end, we propose a novel object tracking algorithm for RGB-T image sequences, namely MaCNet. The overall algorithm structure is shown in Figure 1. Within the framework of deep learning and tracking-by-detection, our tracker includes three modules: feature extraction network, modal-aware attention network, and classification network. The feature extraction network consisting of three convolutional layers takes the form of a two-stream network for independent feature extraction of visible spectrum and thermal infrared modalities. The modal-aware attention network is used for cross-modal information interaction. On one hand, this module exploits the importance of each convolutional layers directly from the raw data to form a modal-aware attention that is sensitive to the scene. On the other hand, the estimated attention is used to guide the fusion of dual-modality features to increase the response of the network to informative features. The classification network is composed of three parallel branches after the feature extraction network, which respectively acts on the RGB path, the thermal infrared path, and the fusion path, and each branch is a binary classification network containing three fully connected layers. In the offline phase, these three branches complete training with competitive learning, which promotes the optimization of the entire network parameters towards the cooperation and complementarity of the dual-modality data. It is worth noting that only the fusion branch is retained during the online tracking phase, and the remaining two branches will be discarded.

In addition, in order to adapt to the specific requirements of video tracking, such as arbitrary types and unfixed semantic categories of the target, we adopt the strategy of multi-domain learning [24] for the offline training. The third fully connected layer of each binary network contains *k* branches, each branch corresponds to a specific domain, and different targets and scene sequences correspond to different domains. For the different domains of the training data, the entire network is divided into two parts: the shared layers and the domain-specific layers. The former part shares the consistent parameters for all domains, while the latter uses the alternate optimization between different domains to complete the training.

### 3.2. Modal-Aware Attention Network

**Figure 2:**
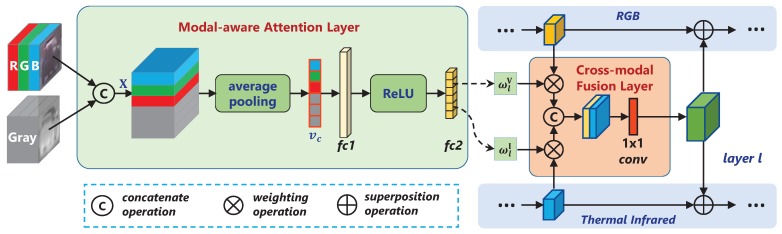
Schematic diagram of modal-aware attention network.

Inspired by the widely adopted attention module [29,30], our modal-aware attention network is dedicated to exploring efficient feature extraction from a cross-modality level. Based on the basic components of the attention module such as the pooling layer, fully connected layer and activation layer, we build a collaborative structure between modalities at different feature layers to achieve informative feature capture with a scene-adaptive manner. As shown in Figure 2, the whole modal-aware attention network is designed into two parts, which are composed of a modal-aware attention layer and a cross-modal fusion layer.

The modal-aware attention layer is composed of an average pooling layer, two fully connected layers, and a ReLU layer. Specifically, the initial sample images of the two modalities are first concatenated into $x\in R^{H\times W\times 2C}$, where *H*, *W* and *C* are its height, width and the number of channels, respectively. Then map x to a new attention space Ω through the modal-aware attention layer, denoted as A, which is given by

(1) $$A\left(x\right)=W_{2}R\left(W_{1}\underset{\mathrm{average}\;\mathrm{pooling}}{\underbrace{\sum_{i=1}^{H}\sum_{j=1}^{W}x\left(i,j,c\right)}}\right),$$

where $W_{1}$ and $W_{2}$ are learnable weight matrices, R(·) represents a ReLU function, and the average pooling operation is used to reflect the overall response characteristics of each channel. To make more efficient use of raw data, we employ a fully connected layer to directly learn the modality attention weights of all feature extraction layers and denote the weights Ω∈Ω as

(2) $$\Omega =\left[\omega_{1}^{m},\cdots ,\omega_{L}^{m}\right],\;m\in \left\{V,I\right\},$$

where *L* is the total number of convolutional layers, V and I represent the visible spectrum and thermal infrared modality, respectively.

The cross-modal fusion layer is used for information interaction between modalities. The weighted feature maps are first concatenated by channel, obtaining

(3) $$c_{l}^{x}=\mathrm{Concat}\left[\omega_{l}^{V}x_{l}^{V},\omega_{k}^{I}x_{l}^{I}\right],\;l\in \left[1,L\right]$$

where $x_{l}^{V}$ and $x_{l}^{I}$ represent the output of the $l^{\mathrm{th}}$ convolutional layer of the two modalities, respectively. In order to fuse the cross-modal information, the fused feature superimpose with each modality feature map is fed to the next convolution layer. This operation can be formalized as

(4) $$x_{l+1}^{m}=\sigma \left(\zeta \left(c_{l}^{x}\right)+x_{l}^{m}\right),\;m\in \left\{V,I\right\},l\in \left[1,L\right],$$

where $x_{l+1}^{m}$ represents the input of convolutional layer l+1 of modality *m*, σ(·) represents a nolinear function. ζ(·) denotes a 1×1 convolution operation, which is used to adjust the cross-modal feature map to the same size as the original convolution feature map of each modality.

### 3.3. Modal Competitive Learning Algorithm

In order to obtain better results than single modality in dual modalities tracking, another key is to establish an effective object appearance model based on the fused features, and to achieve the object and background discrimination in more challenging scenarios. To this end, we introduce a model learning scheme with competition between modalities, which is aimed to guide the entire network to optimize in the direction that the two modalities cooperate and complement each other. After cascading to the feature extraction network, we design three independent classification branches corresponding to RGB features, thermal infrared features, and fusion features, then construct an adversarial loss function based on self-affirmation principle of modality performance. Furthermore, the learning of network parameters is performed in a way that the three classification results compete with each other.

The goal of competitive learning is to achieve a classification loss of the fusion branch that is lower than any single-modality branch. Specifically, we first calculate the cross-entropy loss of three independent binary classification networks, i.e., the corresponding form is

(5) $$L=-\sum_{i=1}^{N}\left(y_{i}\log\left(p(x_{i})\right)+\left(1-y_{i}\right)\log\left(1-p(x_{i})\right)\right)$$

where $\left(x_{i},y_{i}\right)$ represents the *i*-th sample and its label, *N* is the number of samples and p(·) denotes the output of softmax. Based on this loss function definition, the fusion branch uses RGB branch and thermal infrared branch as competitors to conduct competitive learning in the form of self-affirmation. The corresponding loss function with the penalty term is defined as

(6) $$L_{C}^{F}=L^{F}+\underset{\mathrm{penalty}\;\mathrm{term}}{\underbrace{\max\left(L^{F}-L^{V},L^{F}-L^{I},0\right)}},$$

where $L^{V}$, $L^{I}$, and $L^{F}$ represent the basic cross-entropy loss functions as Equation (5) of the RGB branch, the thermal infrared branch, and the fusion branch, respectively. At the same time, both the RGB branch and the thermal infrared branch utilize the fusion branch as an opponent to competitive learning, and their antagonistic loss function can be formalized as

(7) $$L_{C}^{m}=L^{m}+\underset{\mathrm{penalty}\;\mathrm{term}}{\underbrace{\max\left(L^{m}-L^{F},0\right)}}\;m\in \left\{V,I\right\}.$$

It should be noted that the difference between the penalty terms of Equations (6) and (7) is that our purpose is to explore the complementary characteristics between the two modalities, so they are more cooperative than competitive. The fusion branch and the two modality branches are mutually motivated through competition, which helps to find a better feature fusion solution.

Based on the above competitive loss functions, we use a part-by-part iterative training strategy to optimize network parameters. The details of the offline training process will be discussed in Section 4.2 and Section 4.3.

## 4. Implementation

In this section, we mainly describe the implementation of the entire tracker, including network parameters settings, offline training details, and online tracking procedures.

### 4.1. Network Parameters

(1) Feature extraction network. We adopt a more lightweight VGG-M network [23] as the backbone network of our feature extraction module. Considering the difference between the two modalities, we separately train the feature extraction layers of each modality instead of sharing their weights. For clarity and completeness of the MaCNet method description, as shown in Table 1, we briefly introduce the configuration of the feature extraction network below. Specifically, our feature extraction network consists of the first three layers of VGG-M, where the sizes of the convolution kernel are 7×7×96, 5×5×256 and 3×3×512, respectively. Each layer is composed of a convolutional layer, a ReLU activation function, a local response normalization (LRN), and a maximum pooling layer. The network inputs are the candidate patches cropped from the aligned dual-modality images, and they are resampled to the size of 107×107. The initial weights of the two-stream path are transferred from the VGG-M model trained on the large-scale dataset ImageNet [39], and then fine-tuning independently for each branch to adapt to the corresponding modality.

(2) Modal-aware attention network. To adapt to the backbone network structure and ensure the dimensional balance of data among modalities, we extend the thermal infrared image to three channels. The adaptive average pooling operation is performed on the dual-modality data to generate a six-channel pooling vector, and then the 12-node and 6-node fully connected layers are sequentially used to estimate the attention parameters of different convolutional layers. At the same time, the ReLU operation is applied to each node in the first fully connected layer to increase the nonlinear fitting ability of the network and prevent the gradient from disappearing when the node is activated.

(3) Classification network. Three binary classification networks are designed for the visible spectrum, thermal infrared and fusion branches. As shown in Table 2, each branch consists of three fully connected layers with the network hyper-parameters are set to 512, 512 and 2 output units, respectively. We employ the multi-domain learning strategy [24], which treats each semantic definition that divides objects and backgrounds as a domain. The overall network has *k* domain branches, which are denoted by the last fully connected layers and recorded as $\mathrm{fc}6_{1}∼{\mathrm{fc}6}_{k}$.

### 4.2. Offline Training

The training process of the entire network mainly consists of two phases, offline training, and online updating. In this section, we mainly introduce the details of the offline training phase.

The offline training phase can be divided into four steps. First, we employ the pre-trained model of the VGG-M network to simultaneously initialize the weights of three convolutional layers in two modalities. At this time, the fully connected layers in the classification network are randomly initialized. Then, in order to adapt to the dual-modality data, we fix the parameters of the modal-aware attention network and fine-tuned the network path of two modalities separately, i.e., the weights of the feature extraction layer and the classification layer are updated. The learning rates of the convolutional layer in the feature extraction network and the fully connected layer in the classification network are set to 0.0001 and 0.001, respectively, and 100 iterations are performed at this stage. The third step is that we fix all the parameters of the feature extraction network, train the modal-aware attention network with a learning rate of 0.0001, and fine-tune the classification network parameters with a learning rate of 0.0005. The iteration epoch is set to 100. Finally, we utilize competitive learning to conduct adversarial training on the fully connected layers of the three classification branches, while keeping the parameters of the feature extraction network and the modal-aware attention network fixed, where the learning rate is set to 0.001 and 100 iterations are performed. In each iteration, we input a mini-batch of 32 positive samples and 96 negative samples. Note that the weight decay in each of the above steps is 0.0005 and the momentum is set to 0.9.

The training process of the entire network uses the ordinary stochastic gradient descent (SGD) method, and each domain is processed separately in each iteration. The candidate samples are generated by Gaussian sampling whose mean is the ground-truth of target bounding box, and using intersection over union (IoU) overlap ratio of samples and ground-truth bounding boxes as a metric, 50 positive samples (IoU ratio greater than 0.7) and 200 negative samples (IoU ratio less than 0.5) are collected in each frame.

### 4.3. Online Tracking

In the tracking process, we fix all the parameters of the feature extraction network and modal-aware attention network, only fine-tune the network parameters of the fusion classification branch with the same implementation as [24]. For each test sequence, *k* domain branches of the last fully connected layer are replaced by a single branch and updated in the subsequent frame pairs. Specifically, given the first frame pair of the sequence and the ground-truth bounding box, we collect 500 positive samples (IoU ratio with ground-truth greater than 0.7) and 5000 negative samples (IoU ratio with ground-truth less than 0.5) to train a new domain-specific layer. For the last layer and the other two layers of the fully connected layers, the learning rates are set to 0.001 and 0.0005, respectively. We train the new branch with 50 iterations, and the weight decay and momentum are fixed to 0.0005 and 0.9, respectively. In the following frames, we collect positive samples (IoU ratio with ground-truth greater than 0.7) and negative samples (IoU ratio with ground-truth less than 0.3) as training samples for long-term update and short-term update, and the learning rate of the last layer and the other two layers of the fully connected layers are set to 0.002 and 0.0002, respectively.

At frame *t*, we first build a candidate set $\left\{x_{t}^{i}\right\}$ from a Gaussian distribution of previous frame tracking result t−1, where the mean of Gaussian function is the center position of previous frame and the covariance is set to $diag\{0.09r^{2},0.09r^{2},0.25\}$, where *r* is the mean of the width and height of target in the previous frame. Each positive sample (IoU with previous target bounding box greater than 0.6) and negative sample (IoU with previous target bounding box less than 0.3) from the candidate set are fed into our network as the current frame inputs and obtain their classification scores. The positive and negative scores for sample *i* are denoted as $f^{+}\left(x_{t}^{i}\right)$ and $f^{-}\left(x_{t}^{i}\right)$. We sort the samples by scores and select the candidate sample with the highest score as the tracking result $x_{t}^{*}$ of frame *t*, i.e.,

(8) $$x_{t}^{*}=\arg\max_{i}f^{+}\left(x_{t}^{i}\right),\;i=1,\cdots ,256.$$

We also apply a bounding box regression [24] to further improve the localization accuracy and solve the problem of target scale change during the tracking process. It is worth noting that we only train a bounding box regressor in the first frame of each test sequence, so as to avoid the potential unreliability in the subsequent frames.

## 5. Experiments

To validate the effectiveness of the proposed MaCNet, we evaluate it on two popular large-scale RGB-T tracking benchmarks: GTOT dataset [12] and RGBT234 dataset [13]. We compared the performance with the state-of-the-art RGB-T trackers and RGB trackers, and evaluated each major component of MaCNet to analyze their effectiveness. During the experiments, we first train our network using the RGBT234 dataset [13] and test it on the GTOT dataset [12]. In another experiment, We exchange training and test sets, that is, GTOT dataset [12] is used as training data and RGBT234 dataset [13] is used as test data.

### 5.1. Evaluation Setting

(1) Datasets. The GTOT dataset [12] and the RGBT234 dataset [13] are two large-scale RGB-T tracking datasets released in recent years. They are captured by two FOV-aligned cameras and the data content is very challenging. The GTOT dataset [12] has 50 RGB-T video clips with target annotations under different scenes and conditions. To analyze the sensitivity of the RGB-T tracking methods to different attributes, the entire dataset is divided into seven subsets, corresponding to the challenges of different attributes. It contains a total of approximately 15,000 frames, many of which are small targets. RGBT234 dataset [13] is extended from the RGB-T210 dataset [3]. It contains a total of 234 highly aligned RGB-T video pairs. Its total number of frames reaches about 234,000 and the longest video pair length up to 8000 frames. In order to analyze the effectiveness of different tracking algorithms for different challenges, 12 attributes are labeled for RGBT234 dataset [13].

(2) Evaluation metrics. We employ two widely used indicators of precision rate (PR) and success rate (SR) for quantitative performance evaluation. PR is the percentage of video frames whose distance between the center point of the target location estimated by the tracking algorithm and the corresponding ground-truth is less than a given threshold. SR is the percentage between the number of frames in which the overlap ratio of the bounding box obtained by the tracker and its ground-truth is greater than the set threshold and the total number of frames of the video. Different SR plots can be obtained by changing the threshold, and the area under the success rate curves can be used as the representative SR for quantitative performance evaluation. Since the target of the GTOT dataset [12] is relatively small, we set the thresholds to 5 and 20 pixels for GTOT and RGBT234 [13] datasets respectively.

### 5.2. Evaluation on GTOT Dataset

**Figure 3:**
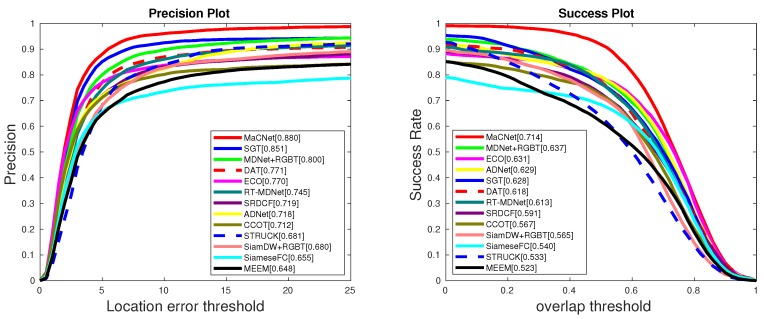
Precision rate (PR) and success rate (SR) curves of different tracking result on GTOT dataset [12], where the representative PR and SR scores are presented in the legend.

(1) Comparison with RGB-T trackers. On the GTOT dataset [12], we compare our method with 12 state-of-the-art trackers, including DAT [27], ECO [25], CCOT [21], MEEM [40], SRDCF [20], SiameseFC [26], ADNet [41], STRUCK [1], RT-MDNet [42], MDNet [24]+RGBT, SiamDW [43]+RGBT and SGT [3]. Since there are fewer existing RGB-T trackers, some RGB approaches extend the RGB-T tracking by concatenating RGB and thermal infrared features into a single vector or by considering the thermal infrared image as one or three additional channels of RGB. In the above trackers, the last three methods are RGB-T based trackers, and the rest are RGB based trackers. Figure 3 shows that our algorithm is obviously better than other state-of-the-art trackers on the GTOT dataset [12], demonstrating the effectiveness of our approach. Specifically, our tracker achieves 8.0%/7.7% and 2.9%/8.6% performance gains in PR/SR over MDNet [24]+RGBT and SGT [3], respectively. In addition, compared with other trackers, our approach also has an obviously superior performance, which shows that our tracker can make good use of RGB and thermal infrared information to construct a robust feature representation and improve tracking performance.

(2) Attribute-based performance. The GTOT dataset [12] contains seven different attributes: occlusion (OCC), large scale variation (LSV), fast motion (FM), low illumination (LI), thermal crossover (TC), small object (SO), and deformation (DEF). To analyze the sensitivity of our MaCNet to different attributes, we also compare it with 12 state-of-the-art algorithms, including DAT [27], ECO [25], SiameseFC [26], MEEM [40], SRDCF [20], ADNet [41], CCOT [21], STRUCK [1], RT-MDNet [42], SGT [3], MDNet [24]+RGBT and SiamDW [43]+RGBT. The results in Table 3 and Table 4 show that our trackers perform best under all other challenges except LSV. One possible reason is that our method is based on a random sampling process of the tracking-by-detection framework, so more candidate samples are required to adapt to the dramatic changes of target scale. ECO [25] constructs a generative model of dense sample space, thereby ensuring the diversity of training samples to obtain a more robust model for LSV attribute.

### 5.3. Evaluation on RGBT234 Dataset

**Figure 4:**
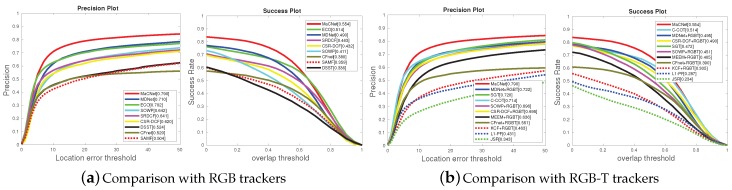
Evaluation results on the RGBT234 [13] benchmark. The representative scores of PR/SR are given in the legend. For a clear comparison, we separately plot the RGB and RGB-T trackers results curves in (**a**) and (**b**).

**Figure 5:**
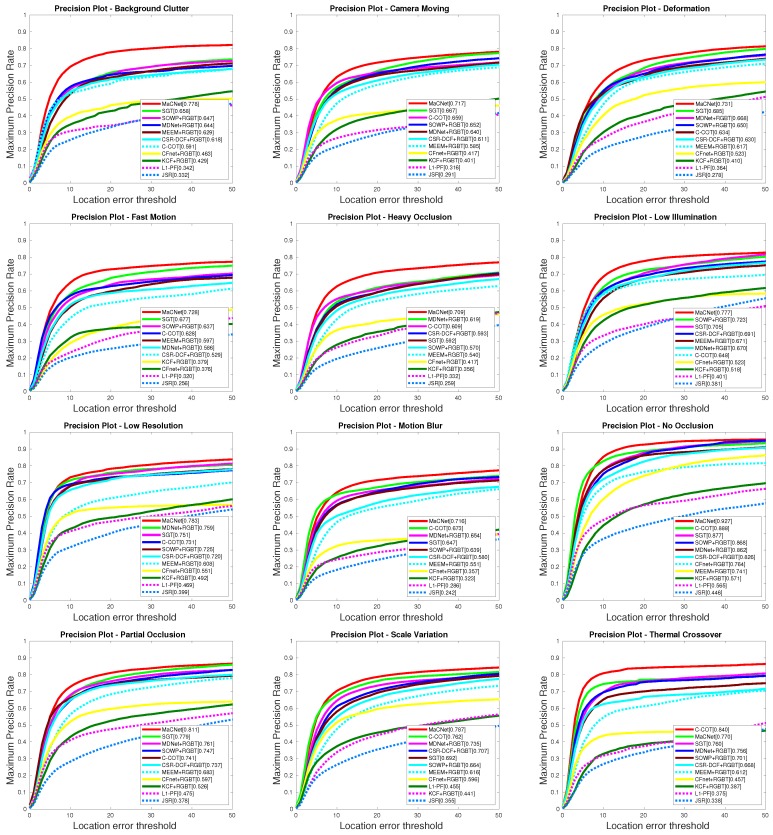
PR evaluation results on various attribute challenges comparing to ten state-of-the-art approaches on RGBT234 [13].

**Figure 6:**
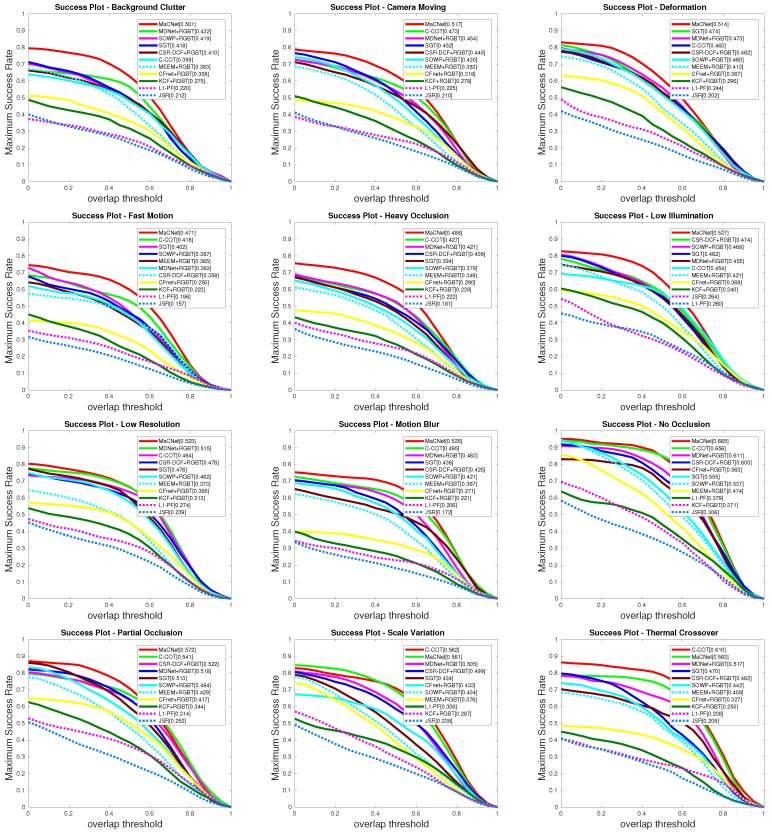
SR evaluation results on various attribute challenges comparing to ten state-of-the-art approaches on RGBT234 [13].

(1) Comparison with RGB trackers. For a more comprehensive evaluation, this experiment validates the superiority of the proposed RGB-T tracking approach over RGB trackers. We evaluated our MaCNet on the RGBT234 dataset [13] with eight state-of-the-art RGB trackers, including MDNet [24], ECO [25], SRDCF [20], SOWP [44], CSR-DCF [22], CFnet [17], DSST [45], SAMF [18]. As illustrated in Figure 4a, our tracker achieves 8.0%/6.4% and 8.8%/4.0% performance gains in PR/SR over MDNet [24] and ECO [25], which demonstrates the necessity of adding thermal infrared information to traditional visual tracking.

(2) Comparison with RGB-T trackers. We also compare our MaCNet versus ten state-of-the-art RGB-T trackers, including MDNet [24]+RGBT, SGT [3], C-COT [21], SOWP [44]+RGBT, CSR-DCF [22]+RGBT, JSR [4], L1-PF [7], MEEM [40]+RGBT, CFnet [17]+RGBT, KCF [19]+RGBT. Figure 4b shows that our tracker is significantly better than them. From the results, our tracker achieves 6.8%/5.9% and 7.0%/8.2% performance gains in PR/SR over MDNet [24]+RGBT and SGT [3], respectively. These results also confirm that our schemes for feature fusion and model training can effectively utilize dual-modality information to construct a more reliable target representation.

(3) Attribute-based performance. We also report the attribute-based results of our MaCNet versus other state-of-the-art eight RGB trackers and ten RGB-T trackers on the RGBT234 dataset [13]. RGB tracking algorithms include: MDNet [24], ECO [25], SRDCF [20], SOWP [44], CSR-DCF [22], CFnet [17], DSST [45], SAMF [18]. RGB-T tracking algorithms include: MDNet [24]+RGBT, SGT [3], C-COT [21], SOWP [44]+RGBT, CSR-DCF [22]+RGBT, JSR [4], L1-PF [7], MEEM [40]+RGBT, CFnet [17]+RGBT, KCF [19]+RGBT. The attributes shown in Table 5 and Table 6 include no occlusion (NO), partial occlusion (PO), heavy occlusion (HO), low illumination (LI), low resolution (LR), thermal crossover (TC), deformation (DEF), fast motion. (FM), scale variation (SV), motion blur (MB), camera moving (CM), and background clutter (BC). From Table 5 and Table 6 we observe that our RGB-T tracker performs better than RGB trackers in most challenges except for TC. Figure 5 displays the results of the prediction rate of our tracker and other RGB-T trackers on each attributes of the RGBT234 dataset [13], and the success rate results are shown in Figure 6. From these PR/SR results, we observe that our MaCNet can well handle various challenging factors and outperform the other trackers in ten out of twelve attributes.

### 5.4. Ablation Study

To verify the effectiveness of each major component of MaCNet, we compare the following three algorithm variants on the GTOT dataset [12]. (1) MaCNet-noMAA eliminates the modal-aware attention network and uses only feature extraction layers and classification layers for tracking. (2) MaCNet-noCL removes the competitive learning loss and uses only the standard cross-entropy binary classification loss. (3) Only-pretrain uses the RGB-T dataset for fine-tuning after we load the VGG-M [23] parameters, primarily to demonstrate the necessity of adapting to the RGB-T dataset.

**Figure 7:**
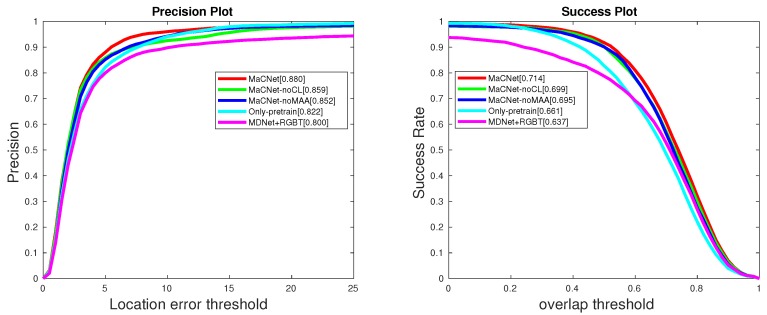
The comparison results of MaCNet and its variants on the GTOT dataset [12], where the representative PR and SR scores are presented in the legend.

From the results of Table 7 and Figure 7, we can draw the following conclusions. (1) MaCNet is significantly better than MaCNet-noMAA, which indicates that the modal-aware attention network can better consider the importance of the information provided by each modality in feature extraction. (2) MaCNet outperforms MaCNet-noCL, which is a good illustration of the importance of considering heterogeneity and complementarity between modalities in multimodal tasks. Competitive learning can better integrate the complementary information provided by the two modalities to improve tracking performance. (3) The result of Only-pretrain is better than MDNet [24]+RGBT, which indicates that it is necessary to fine-tune the parameters of the feature extraction network using the RGB-T dataset in the pre-training phase.

### 5.5. Efficiency Analysis

**Figure 8:**
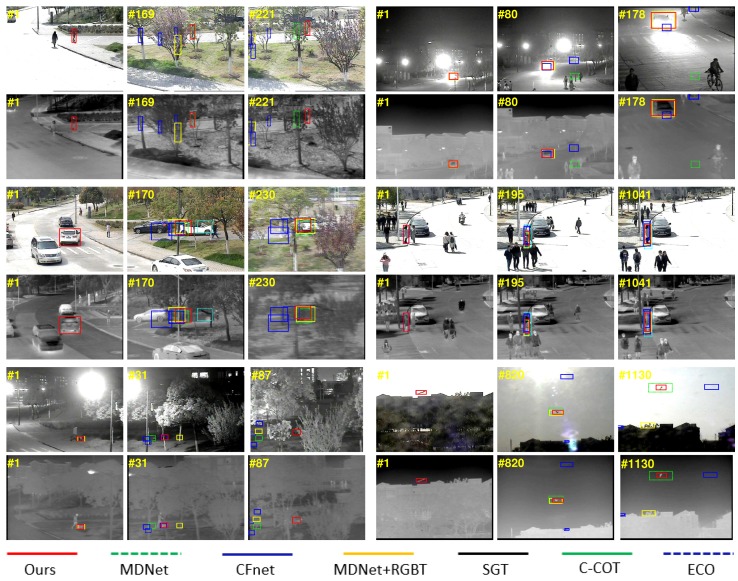
Qualitative comparison of our MaCNet versus three state-of-the-art RGB trackers and three state-of-the-art RGB-T trackers on six video sequences.

We implemented our approach on the PyTorch platform with 3.6 GHz Intel Core i7-6850K CPU, NVIDIA GeForce GTX 1080 Ti GPU and 16 G RAM. The frames rate of our MaCNet is approximately 0.8 FPS, while MDNet [24]+RGBT is 1.6 FPS. Note that MDNet [24]+RGBT is tracked by running a thermal infrared image into the MDNet [24] as an additional channel for the RGB image. Figure 8 shows a qualitative comparison of our algorithm with three state-of-the-art RGB trackers and three state-of-the-art RGB-T trackers on partial video sequences, including MDNet [24], ECO [25], CFnet [17], MDNet [24]+RGBT, SGT [3] and C-COT [21]. In general, our approach shows better performance in dealing with challenges such as partial occlusion, motion blur, background clutter, illumination variations, low resolution, and large appearance changes. It also intuitively demonstrates the effectiveness of our approach.

## 6. Conclusions

In this paper, we propose a MaCNet algorithm based on a modal-aware attention network and competitive learning, which is an object tracking approach for RGB-T dual-modality data. The method evaluates the modality importance of different scenes through the modal-aware attention module and achieves an adaptive fusion for multi-level features between modalities. Moreover, through introducing a competitive learning strategy, a better-performing feature fusion method and classifier are trained to achieve a cooperative and complementary representation of infrared and visible spectral data. A large number of experiments on the public datasets demonstrate the effectiveness of the algorithm for dual-modality data mining and utilization. In the future work, we will investigate a deeper and wider network to enhance feature representation and further improve RGB-T tracking performance, and use similar feature pruning to eliminate redundancy and unnecessary calculations and achieve real-time object tracking.

## Figures and Tables

**Table 1:** The configuration of feature extraction network and architecture details.

Layers	Kernel Size	Stride	Channels
(input)			×3
conv-1	7×7	2	×96
ReLU			×96
LRN			×96
MaxPool	3×3	2	×96
conv-2	5×5	2	×256
ReLU			×256
LRN			×256
MaxPool	3×3	2	×256
conv-3	3×3	2	×512
ReLU			×512

**Table 2:** The configuration of classification network and architecture details.

RGB Branch	Fusion Branch	Thermal Infrared Branch
	Dropout	
Linear (fc4)	Linear (fc4)	Linear (fc4)
ReLU	ReLU	ReLU
Dropout	Dropout	Dropout
Linear (fc5)	Linear (fc5)	Linear (fc5)
ReLU	ReLU	ReLU
Dropout	Dropout	Dropout
Linear (fc6)	Linear (fc6)	Linear (fc6)
softmax	softmax	softmax

**Table 3:** Attribute-based PR scores (%) on GTOT dataset [12] against with twelve RGB-T trackers. Optimal and suboptimal results are represented by red and green fonts, respectively.

Attributes	OCC	LSV	FM	LI	TC	SO	DEF	ALL
MEEM [40]	68.4	62.8	68.6	66.5	69.6	69.3	68.8	64.8
SiameseFC [26]	70.2	78.7	72.7	61.5	74.7	72.4	53.8	65.5
SiamDW [43]+RGBT	67.5	68.9	71.1	70.0	63.5	76.4	69.1	68.0
STRUCK [1]	67.4	66.0	64.0	74.0	68.0	74.5	75.6	68.1
CCOT [21]	75.1	81.8	75.4	71.3	74.5	83.8	66.4	71.2
ADNet [41]	72.7	74.8	72.8	71.5	71.2	81.9	70.7	71.8
SRDCF [20]	72.7	80.4	68.3	71.7	70.5	80.5	66.6	71.9
RT-MDNet [42]	73.3	79.1	78.1	77.2	73.7	85.6	73.1	74.5
ECO [25]	77.5	85.6	77.9	75.2	81.9	90.7	75.2	77.0
DAT [27]	77.2	78.6	82.0	76.0	80.9	88.6	76.9	77.1
MDNet [24]+RGBT	82.9	77.0	80.5	79.5	79.5	87.0	81.6	80.0
SGT [3]	81.0	84.2	79.9	88.4	84.8	91.7	91.9	85.1
MaCNet	87.6	84.6	82.3	89.4	89.2	95.0	92.6	88.0

**Table 4:** Attribute-based SR scores (%) on GTOT dataset [12] against with twelve RGB-T trackers. Optimal and suboptimal results are represented by red and green fonts, respectively.

Attributes	OCC	LSV	FM	LI	TC	SO	DEF	ALL
MEEM [40]	53.0	46.2	52.3	51.8	54.6	49.6	57.8	52.3
SiameseFC [26]	55.9	63.5	60.4	50.7	59.5	55.2	45.0	54.0
SiamDW [43]+RGBT	53.6	56.5	57.6	58.8	51.7	58.8	58.2	56.5
STRUCK [1]	51.6	49.6	51.8	55.3	51.0	52.7	60.4	53.3
CCOT [21]	57.6	66.2	61.0	56.3	57.9	59.9	51.6	56.7
ADNet [41]	60.0	63.6	60.6	64.4	59.9	63.7	63.2	62.9
SRDCF [20]	58.0	68.1	61.1	59.4	58.0	57.5	53.7	59.1
RT-MDNet [42]	57.6	63.7	64.1	63.8	59.0	63.4	61.0	61.3
ECO [25]	62.2	70.5	64.5	61.7	65.3	69.1	59.8	63.1
DAT [27]	59.2	62.4	61.5	60.9	62.6	64.4	63.3	61.8
MDNet [24]+RGBT	64.1	57.3	59.8	64.3	60.9	62.2	68.8	63.7
SGT [3]	56.7	54.7	55.9	65.1	61.5	61.8	73.3	62.8
MaCNet	68.7	67.3	65.9	73.1	69.7	69.5	76.5	71.4

**Table 5:** Attribute-based PR scores (%) on RGBT234 dataset [13] against with eight RGB trackers. Optimal and suboptimal results are represented by red and green fonts, respectively.

Attributes	NO	PO	HO	LI	LR	TC	DEF	FM	SV	MB	CM	BC	ALL
SAMF [18]	67.6	54.0	39.8	46.8	50.7	54.7	42.4	42.4	55.4	37.8	40.2	37.6	50.4
CFnet [17]	72.4	57.7	37.9	43.6	48.2	51.2	46.0	36.3	59.5	38.4	41.7	36.3	52.0
DSST [45]	69.7	56.5	41.0	48.3	57.9	49.5	43.8	35.5	56.8	35.8	39.9	45.8	52.4
CSR-DCF [22]	78.8	64.1	52.2	49.0	57.9	62.9	55.7	53.0	67.0	55.0	55.8	50.3	62.0
SRDCF [20]	79.1	68.8	52.6	57.5	62.0	66.1	56.3	52.6	70.4	55.9	56.9	48.1	64.1
SOWP [44]	80.1	66.6	54.7	52.4	67.9	71.2	61.1	57.9	66.6	59.8	59.8	52.8	64.2
ECO [25]	88.0	72.2	60.4	63.5	68.7	82.1	62.2	57.0	74.0	68.9	63.9	57.9	70.2
MDNet [24]	81.2	74.7	63.3	58.9	66.0	74.8	66.4	63.2	73.9	62.4	61.3	62.5	71.0
MaCNet	92.7	81.1	70.9	77.7	78.3	77.0	73.1	72.8	78.7	71.6	71.7	77.8	79.0

**Table 6:** Attribute-based SR scores (%) on RGBT234 dataset [13] against with eight RGB trackers. Optimal and suboptimal results are represented by red and green fonts, respectively.

Attributes	NO	PO	HO	LI	LR	TC	DEF	FM	SV	MB	CM	BC	ALL
SAMF [18]	43.3	38.0	28.5	32.7	32.9	38.1	33.2	27.0	39.7	27.9	30.6	25.9	35.9
CFnet [17]	54.5	41.8	27.2	31.5	33.9	38.5	34.0	25.3	43.2	29.4	32.1	25.7	38.0
DSST [45]	43.3	36.2	27.0	29.9	36.8	32.5	32.5	22.4	33.7	25.1	27.9	29.3	33.6
CSR-DCF [22]	56.6	44.4	36.0	32.9	37.0	42.8	41.0	35.0	47.3	39.8	39.6	32.4	43.2
SRDCF [20]	58.5	49.9	37.1	40.9	41.0	46.7	40.6	34.3	51.8	41.5	40.9	32.4	46.3
SOWP [44]	50.2	42.7	35.4	33.6	42.1	46.2	42.0	33.5	39.6	39.9	39.0	33.6	41.1
ECO [25]	65.5	53.4	43.2	45.0	46.4	60.9	45.8	39.5	55.8	52.3	47.7	39.9	51.4
MDNet [24]	59.0	50.9	43.2	39.6	44.5	53.0	46.8	39.3	51.9	44.2	43.3	41.8	49.0
MaCNet	66.5	57.2	48.8	52.7	52.3	56.3	51.4	47.1	56.1	52.5	51.7	50.1	55.4

**Table 7:** PR/SR scores (%) of different variants induced from modal-aware attention network and competitive learning (MaCNet) on the GTOT dataset [12].

	Only-Pretrain	MaCNet-noMAA	MaCNet-noCL	MaCNet
PR	82.2	85.2	85.9	88.0
SR	66.1	69.5	69.9	71.4
